# Aβ and Inflammatory Stimulus Activate Diverse Signaling Pathways in Monocytic Cells: Implications in Retaining Phagocytosis in Aβ-Laden Environment

**DOI:** 10.3389/fncel.2016.00279

**Published:** 2016-12-05

**Authors:** Ekaterina Savchenko, Tarja Malm, Henna Konttinen, Riikka H. Hämäläinen, Cindy Guerrero-Toro, Sara Wojciechowski, Rashid Giniatullin, Jari Koistinaho, Johanna Magga

**Affiliations:** ^1^Department of Neurobiology, A.I.Virtanen Institute for Molecular Sciences, University of Eastern FinlandKuopio, Finland; ^2^Department of Pharmacology and Toxicology, Research Unit of Biomedicine, University of OuluOulu, Finland

**Keywords:** Alzheimer’s disease, amyloid beta, bone marrow, calcium, cytokines, hematopoietic stem cells, monocytes, phagocytosis

## Abstract

**Background**: Accumulation of amyloid β (Aβ) is one of the main hallmarks of Alzheimer’s disease (AD). The enhancement of Aβ clearance may provide therapeutic means to restrict AD pathology. The cellular responses to different forms of Aβ in monocytic cells are poorly known. We aimed to study whether different forms of Aβ induce inflammatory responses in monocytic phagocytes and how Aβ may affect monocytic cell survival and function to retain phagocytosis in Aβ-laden environment.

**Methods**: Monocytic cells were differentiated from bone marrow hematopoietic stem cells (HSC) in the presence of macrophage-colony stimulating factor. Monocytic cells were stimulated with synthetic Aβ42 and intracellular calcium responses were recorded with calcium imaging. The formation of reactive oxygen species (ROS), secretion of cytokines and cell viability were also assessed. Finally, monocytic cells were introduced to native Aβ deposits *ex vivo* and the cellular responses in terms of cell viability, pro-inflammatory activation and phagocytosis were determined. The ability of monocytic cells to phagocytose Aβ plaques was determined after intrahippocampal transplantation *in vivo*.

**Results**: Freshly solubilized Aβ induced calcium oscillations, which persisted after removal of the stimulus. After few hours of aggregation, Aβ was not able to induce oscillations in monocytic cells. Instead, lipopolysaccharide (LPS) induced calcium responses divergent from Aβ-induced response. Furthermore, while LPS induced massive production of pro-inflammatory cytokines, neither synthetic Aβ species nor native Aβ deposits were able to induce pro-inflammatory activation of monocytic cells, contrary to primary microglia. Finally, monocytic cells retained their viability in the presence of Aβ and exhibited phagocytic activity towards native fibrillar Aβ deposits and congophilic Aβ plaques.

**Conclusion**: Monocytic cells carry diverse cellular responses to Aβ and inflammatory stimulus LPS. Even though Aβ species cause specific responses in calcium signaling, they completely lack the ability to induce pro-inflammatory phenotype of monocytic cells. Monocytes retain their viability and function in Aβ-laden brain.

## Introduction

Microglia, the immune cells in the central nervous system (CNS), actively survey their microenvironment in the brain parenchyma. After injury, microglia motility switches from untargeted to targeted movement towards the lesion site (Nimmerjahn et al., 2005). Microglia are thus important regulators of normal brain physiology and also crucial mediators of pathological processes in the CNS (Kettenmann et al., 2011). In Alzheimer’s disease (AD), microglia accumulate around amyloid β (Aβ) deposits, interact with Aβ and possibly contribute to Aβ removal. Many studies indicate that microglia may be renewed from peripheral monocytic cells. The extent and conditions of peripheral monocytic cell migration in AD, however, remain controversial (Malm et al., 2005; Simard et al., 2006; Ajami et al., 2007; Mildner et al., 2007, 2011; Lebson et al., 2010; Hao et al., 2011; Naert and Rivest, 2012; Koronyo et al., 2015). Monocytes, however, may also participate in Aβ removal by not entering the brain parenchyma (Michaud et al., 2013).

The hallmarks of AD include accumulation of Aβ in the brain regions that serve cognition and memory. In the amyloidogenic pathway, amyloid precursor protein is cleaved into Aβ peptides Aβ40 and Aβ42, the latter being more prone to aggregation (De Strooper, 2010). Monomeric Aβ spontaneously aggregates into oligomers and high-molecular weight oligomers, which further aggregate to form insoluble fibrils and eventually, Aβ plaques. Aβ-mediated neurotoxicity is dependent on Aβ aggregation state from which Aβ oligomers are the most neurotoxic as reviewed in Aguzzi and O’Connor (2010), and De Strooper (2010). Furthermore, Aβ signaling and Aβ removal by astrocytes and microglia are also dependent on the Aβ aggregation state (D’Andrea et al., 2004; Mandrekar et al., 2009; Michelucci et al., 2009; Sondag et al., 2009; Nielsen et al., 2010).

The late-onset AD is associated with a 30% decrease in the clearance of Aβ (Mawuenyega et al., 2010) which may eventually lead to accumulation of Aβ and disturbance of neuronal homeostasis. In genome wide analysis, many susceptibility loci associated with microglial and myeloid cell function have been identified for late-onset AD (Guerreiro et al., 2013; Lambert et al., 2013; Benitez et al., 2014; Ramanan et al., 2015). Since proper Aβ clearance may play a crucial role in the development of AD, the mechanisms of Aβ clearance are under intensive investigation also in terms of therapeutic aspects. The migration of monocytic cells may require specific factors and the infiltration of these cells into the brain in sufficient quantities may be a challenge. We have provided a method which allows genetic manipulation of hematopoietic stem cells (HSC) and their subsequent *in vitro* differentiation into phagocytic monocytic cells (Magga et al., 2012). By utilizing these cells as a model, we investigated the cellular responses of monocytic cells into different species of Aβ in terms of cellular signaling, cytokine production, reactive oxygen species (ROS) and nitric oxide (NO) production, phagocytosis of Aβ and cell viability. We demonstrate that opposite to inflammatory stimulus induced by lipopolysaccharide (LPS), Aβ species completely lack pro-inflammatory activation of monocytic cells, contrary to that observed in primary microglia. Instead, freshly solubilized Aβ induces calcium oscillations and a minor production of anti-inflammatory cytokine interleukin-10 (IL-10). In addition, monocytic cells retain their function and characteristics as phagocytic cells in the brain with native Aβ plaques.

## Materials and Methods

### Cell Culture

Monocytic cells were cultivated as described before Magga et al. (2012). Briefly, bone marrow was isolated from 6- to 8-week-old C57BL mice. When needed to obtain greater amount of HSCs, or to obtain HSCs from mice over 8-weeks-old, adult mice were treated s.c. with a single dose of granulocyte colony stimulating factor 500 μg/kg (Pegfilgrastim, Neulasta, Amgen, diluted in sterile 0.15 M sodium acetate, pH adjusted to 7.4. with acetic acid) 3–4 days prior to the sacrifice to mobilize HSCs. Then, bone marrow mononuclear cells were isolated by gradient centrifugation with Ficoll paque (GE Healthcare) and HSCs were isolated by immunomagnetic cell separation using CD117 mouse HSC positive selection kit (EasySep, StemCell Technologies). CD117^+^ cells were plated at 100,000 cells/cm^2^ and proliferated in serum-free conditions in a humidified atmosphere at 37°C in 5% CO_2_ as described (Malm et al., 2008). Non-adherent cells were replated every 2 days when half of the medium was refreshed. For differentiation, non-adherent cells were collected and plated at 100,000 cells/cm^2^ in Iscove’s modified Dulbecco’s medium (IMDM) in the presence of low endotoxin serum, L-glutamine, penicillin-streptomycin (all products from Gibco, Thermo Fisher Scientific, Waltham, MA, USA), 100 μM β-mercaptoethanol (Sigma) and 10 ng/ml macrophage colony stimulating factor (MCSF; R&D Systems, Minneapolis, MN, USA). After differentiation, cells were collected in PBS when needed.

Primary mouse postnatal day P0-P1 microglia cultures were prepared from cortices and hippocampi and cultivated as a mixed astrocyte/microglia culture as described before Magga et al. (2012). Nonadherent microglia present above the astrocyte layer were collected by shaking the plates 10–15 min at 120 rpm at 37°C and collection of supernatant. Adherent microglia below the astrocyte layer were collected by removal of astrocyte layer with mild trypsinization and collection of remaining microglia from bottom of the flask with repeated pipetting in PBS, as described earlier (Magga et al., 2012). After collection and when plated as microglia culture, both cell types adhered to surface. Microglia were cultivated in IMDM, 10% low endotoxin serum, L-glutamine, penicillin-streptomycin (all products from Gibco, Thermo Fisher Scientific, Waltham, MA, USA).

### Aβ Preparation

Aβ species were prepared as described before Magga et al. (2010). Aβ42 (American Peptide) was dissolved into a stock solution of 1 mg/ml in sterile water (soluble Aβ termed as “sAβ”). To obtain fully fibrillized Aβ (termed as “fAβ”), the dissolved peptide was incubated at 37°C for a week. We have previously analyzed these Aβ preparations with immunoblotting for human Aβ (clone 6E10, Signet, Covance) after cross-linking the samples with glutaraldehyde (Sigma; Kanninen et al., 2008; Magga et al., 2010). Immediately after dissolving the peptide, the Aβ42 peptide preparation contained monomers, dimers, various forms of oligomers and large high molecular weight aggregates. Within time, the amount of low molecular weight forms decreased, as analyzed 24 h to 48 h after solubilization. In *in vitro* studies, we used the concentration of Aβ42 preparation which has been neurotoxic in our previous studies in primary hippocampal neurons and neural stem cells, including dose-response assays for cell viability and neural stem cell migration (Kanninen et al., 2008; Karkkainen et al., 2012; Kärkkäinen et al., 2014).

To obtain native Aβ deposits (termed as “native Aβ”), brains were excised from aged APdE9 mice (Jankowsky et al., 2004) overexpressing human amyloid precursor protein APP695 Swedish mutation and human presenilin-1 deletion in exon 9 (dE9) genes. Brains were frozen on dry ice and processed as previously described (Pihlaja et al., 2008; Magga et al., 2010). Briefly, cryostat-cut 10-μm-thick sagittal brain sections were mounted on glass coverslips and transferred onto 48-well cell culture plates and stored at −20°C until used. Brain sections were thawed shortly prior to the use and rehydrated with cell culture medium before application of cells on top of the sections.

### Calcium Imaging

Cells were differentiated for 2 days, and then replated in differentiation medium onto 25-mm glass coverslips (Merck) for another 3 days. Calcium imaging was performed as described previously (Magga et al., 2006). Briefly, cells were loaded with 4 μM fura-2 acetoxymethyl ester (Molecular Probes) at 37°C for 20 min in HEPES-buffered medium (137 mM NaCl, 5 mM KCl, 0.44 mM KH_2_PO_4_, 4.2 mM NaHCO_3_, 10 mM glucose, 20 mM HEPES, 1 mM probenecid, 1 mM CaCl_2_ and 1.2 mM MgCl_2_ (all reagents from Sigma), pH 7.4). The coverslip was attached to the bottom of a 37°C thermostated perfusion chamber and stimulants were applied by perfusion in HEPES-buffered medium. The cells were excited by alternating 340 and 380 nm UV light with a filter exchange in the control of an InCyt2 system (Intracellular Imaging) and emission was measured through a dichroic mirror and a 510 nm barrier filter with a Cohu CCD camera. A new ratio image (340/380 nm) was collected every second. Primary microglia were applied to calcium imaging 2–3 days after their plating to coverslips. Microglia were labeled with 2 μM fluo-3 acetoxymethyl ester and imaged with TillVision equipped with rapid shifting monochromator Polychrom V with wavelength 488 nm (TILL Photonics GmbH) and perfused with HEPES-buffered medium. Cells were viewed via Olympus IX-70 microscope with specific filter using 10× objective. Images were collected using Sensicam VGA digital camera (PCO AG) at sampling frequency of two frames per second. The data were analyzed with Origin (OriginLab) for quantification of responsive cells and their peak amplitude. The cell was determined viable if it responded to any of the stimuli used.

### Cytokine Assay

After 2 days of differentiation, cells were replated onto 24-well plate at 70,000 cells/cm^2^ for another 2 days. Cells were cultivated in differentiation medium and treated with 50 ng/ml LPS (from *Escherichia Coli*, serotype O111:B4, Sigma) or 20 μM sAβ or 20 μM fAβ. After 24 h of incubation, media were collected, centrifuged to remove any cell debris, and the supernatant was frozen at −70°C. Cytokine concentrations were determined with tumor necrosis factor α (TNFα) enzyme-linked immunosorbent assay (ELISA; R&D Systems, Minneapolis, MN, USA). Cytokine production was also determined from cell culture medium collected after 100 ng/ml LPS, 10 μM sAβ or 10 μM fAβ stimulation. Cytokines were analyzed with cytometric bead array mouse inflammation kit (BD Biosciences) according to manufacturer’s instructions. Primary microglia were treated with stimulants 2 days after their separation from astrocyte co-culture. Cytokine production was analyzed from medium with cytometric bead array kit.

### Nitric Oxide Assay

After 2 days of differentiation, cells were replated onto 96-well plate at 100,000 cells/cm^2^ for another day. Cells were cultivated in phenol red–free differentiation medium and treated with 100 ng/ml LPS, 10 μM sAβ, 10 μM fAβ or 300 μM paraquat (Sigma). After 24 h of incubation, media were collected and mixed with an equal volume of Griess reagent (2% phosphoric acid, 1% sulfanilamide, 0.1% naphthylethylene dihydrochloride; Sigma), incubated for 10 min. Absorbance was measured at 540 nm with a multiscan reader. Sodium nitrite (Sigma) was used as a standard.

### ROS Assay

After 2 days of differentiation, cells were replated onto 96-well plate at 100,000 cells/cm^2^ for another day. Cells were cultivated in differentiation medium and treated with 100 ng/ml LPS, 10 μM sAβ, 10 μM fAβ or 300 μM paraquat (Sigma). After 4 h of incubation, medium was removed and replaced with ROS assay buffer (HBSS, 15 mM HEPES, 5.5 mM glucose and 1.8 mM CaCl_2_, Sigma) supplemented with 20 μM 2’7’-dichlorofluorescein (Sigma) and incubated at 37°C for 30 min. Thereafter, cells were washed once with ROS assay buffer and ROS production was measured with excitation 485 nm, emission 535 nm with multi-label microplate reader (Victor Wallac).

### Cell Viability Assay

After 2 days of differentiation, cells were replated onto 48-well plate as 100,000 cells/cm^2^ for another day. Cells were cultivated in differentiation medium and treated with 100 ng/ml LPS, 10 μM sAβ, 10 μM fAβ or 300 μM paraquat (Sigma). After 24 h of incubation, 10 μM resazurin (Sigma) was applied and incubated for 4 h. Medium samples were collected into a 96-well plate and measured by excitation at 544 nm and emission at 590 nm on a multiplate reader (Victor Wallac).

### Cell Migration Assay

Cell migration was analyzed by live cell imaging system Cell-IQ, equipped with a phase-contrast microscope and a camera (Chip-Man Technologies, Tampere, Finland). Monocytic cells growing on a 48-well plate were left untreated or treated with either 100 ng/ml LPS, 10 μM sAβ or 10 μM fAβ and cultured in the Cell-IQ incubator for 12 h. Eight replicate wells were analyzed per treatment. Time-lapse images were taken every 15 min and the trajectory and the speed of cell migration were analyzed for 20 cells per well with Cell-IQ Analyzer software. The total number of analyzed cells per group is 160.

### Flow Cytometry

Cells were counted and stained as described (Malm et al., 2005). Briefly, cells were fixed with 4% paraformaldehyde (Sigma) for 20 min at room temperature and then permeabilized with 0.05% saponin (Sigma) and stained with CD68 (Serotec). A minimum of 10,000 events were acquired on FACSCalibur flow cytometer equipped with a 488 laser (BD Biosciences). Data analysis was performed using Cellquest Pro software (BD Biosciences).

### *Ex Vivo* Aβ Degradation Assay

The cells were incubated in differentiation medium on top of brain sections obtained from aged APdE9 mice containing native Aβ deposits. After 5 days of incubation, Aβ quantification was performed with Aβ ELISA or pan-Aβ immunostaining (both from Biosource) as described (Malm et al., 2008).

### Transplantation

Intrahippocampal transplantation was performed as described (Magga et al., 2012). Briefly, 2-year-old transgenic APdE9 mice and their non-transgenic littermates were anesthetized with isoflurane (Baxter) and placed in a stereotaxis apparatus. Monocytic cells (300,000 in 1 μl of HBSS, 2% fetal bovine serum (both from Gibco, Thermo Fisher Scientific, Waltham, MA, USA)) were injected with a Hamilton syringe into the right hippocampus and the same amount of vehicle was injected into the left hippocampus according to following coordinates: ±0.25 mm medial/lateral, 0.27 mm anterior/posterior, 0.25 mm dorsal/ventral from bregma. Brains were excised 4 days post-transplantation and processed as described (Magga et al., 2012). All animal experiments were conducted according to the national regulations for the use and welfare of laboratory animals and approved by the Animal Experiment Committee in State Provincial Office of Southern Finland.

### Histological Analysis

Histological analysis was performed as described (Malm et al., 2005). Briefly, 20-μm coronal sections were stained with Anti-pan Aβ (rabbit polyclonal, Biosource) or Congo red (Sigma), which was applied as 0.2% Congo red in 0.01% NaOH, saturated NaCl/EtOH solution. Stainings were visualized with fluorescence or light microscope and immunoreactive area was quantified using Image Pro Plus software (Media Cybernetics) from the hippocampal region.

### Statistical Analysis

The data are expressed as mean ± standard deviation and analyzed with SPSS software using Student’s *t*-test or one-way analysis of variance when appropriate, followed by Tukey’s *post hoc* test. The occurrence of calcium responsiveness was evaluated with Fisher’s exact test. *p < 0.05, **p < 0.01, ***p < 0.001.

## Results

### Inflammatory Stimulus and Growth Factor Induce Different Calcium Responses in Monocytic Cells

**Figure 1:**
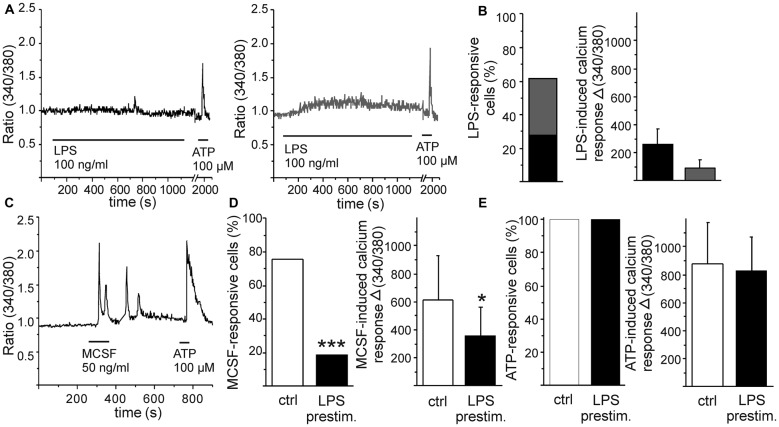
**Pro-inflammatory stimulus lipopolysaccharide (LPS) and growth factor macrophage colony stimulating factor (MCSF) induce distinct calcium responses in monocytic cells.** Monocytic cells were differentiated from hematopoietic stem cells (HSC) in the presence of MCSF for 5 days. Pro-inflammatory stimulus LPS induced a minor elevation in intracellular calcium, either transient (**A**, left panel, marked with black) or slow elevation of the intracellular calcium level (**A**, right panel, marked with gray). Majority of cells responded to LPS (**B**, *n* = 55), but the amplitudes of LPS-induced responses were low (**B**, *n* = 34). Growth factor MCSF induced an oscillatory calcium response **(C)**. LPS stimulation reduced subsequent MSCF-induced calcium response **(D)**, both the number of MCSF-responsive cells (*p* < 0.001, *n* = 71) and the amplitude of MCSF-induced calcium response (*p* < 0.05, *n* = 54). As comparison, LPS had no effect on adenosine triphosphate (ATP)-induced elevation of intracellular calcium, used as an internal control **(E)**. *p < 0.05, ***p < 0.001.

Monocytic cells were differentiated from HSCs in the presence of MCSF. After a few days of differentiation, these cells represent phagocytic activity as well as response to inflammatory stimulus LPS in terms of increased cytokine production and NO release (Magga et al., 2012). Herein, we aimed to further characterize their response to specific inflammatory stimulus induced by Aβ. First, we exposed monocytic cells to a well-known pro-inflammatory stimulus LPS and measured cellular calcium responses by intracellular calcium imaging. After loading the cells with a fluorescent calcium indicator Fura-2, the cells were perfused with a stimulant and individual cells were imaged for determination of intracellular calcium response. LPS induced a minor elevation in intracellular calcium in monocytic cells. This elevation was either a transient elevation (in 46% of the responsive cells) or slow elevation on the in intracellular calcium level (in 54% of the responsive cells; Figures 1A,B). Both the transient elevation and the low elevation in intracellular calcium level were responses of low amplitude (Figures 1A,B). This minor LPS-induced calcium elevation was accompanied with activation of cell mobility. Monocytic cells were static, but within minutes after LPS application they changed into exploring cells. LPS induced monocytic cell protrusions to elongate to random direction in a non-organized manner (experimental observation, data not shown).

MCSF is a differentiation and growth factor for monocytes (Ginhoux and Jung, 2014). When stimulated with a recombinant MCSF, monocytic cells responded with a transient calcium elevation or with an oscillatory calcium response (Figure 1C). As seen in Figure 1A, LPS induced only a minor calcium response in monocytic cells. LPS stimulation, however, reduced both the occurrence and amplitude of subsequent MCSF-induced calcium response either when LPS was inducing a calcium response of its own or when LPS induced no detectable elevation in intracellular calcium at all (Figure 1D). As comparison, LPS had no effect on adenosine triphosphate (ATP)-induced elevation of intracellular calcium (Figure 1E). This indicates that inflammatory stimulus may specifically interfere with monocytic growth factor signaling while leaving at least some other cellular signaling pathways intact. We have previously shown that omission of MCSF reduces monocytic cell viability but if stimulated with LPS, the cell survival increases back to normal level (Magga et al., 2012). LPS and MCSF may thus induce some competitive cellular pathways leading to enhancement of monocytic proliferation/survival. On the other hand, ATP serves as a potential danger signal in CNS and activates microglia. LPS and ATP-mediated signaling may both induce similar types of signals, i.e., microglial motility and the release of various biologically active substances, such as cytokines and inflammatory proteins (Kettenmann et al., 2011). Since we did not observe an overlap in LPS and ATP signaling, they probably utilize different calcium signaling pathways in monocytic cells.

### Soluble Aβ Induces Calcium Oscillations in Monocytic Cells

**Figure 2:**
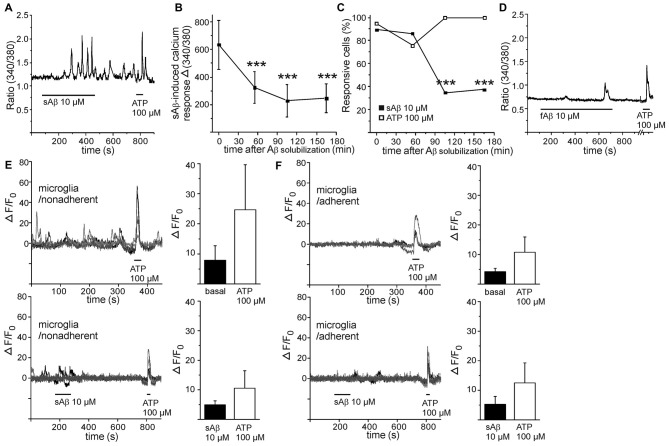
**Soluble amyloid β (Aβ) induces calcium oscillations in monocytic cells.** Freshly solubilized soluble Aβ (sAβ) induced persistent oscillatory calcium responses in monocytic cells **(A)**. Both the amplitude **(B)** and the occurrence of calcium response **(C)** induced by sAβ decayed after the solubilization event time-dependently (*p* < 0.001, *n* = 34). The oscillations induced by sAβ could persist even the maximum recording time, 30 min. As comparison, calcium responses to ATP in the same cells remained constant (**C**, *n* = 38). Fibrillar Aβ (fAβ) induced only a minor calcium response, if any, in monocytic cells **(D)**. Five representative calcium imaging traces shown from microglia collected from astrocyte/microglia coculture above astrocyte layer, termed as nonadherent **(E)** and microglia collected below astrocyte layer, termed as adherent **(F)**, without (upper panels) and with sAβ treatment (lower panels). ATP was used as an intrinsic control. sAβ did not increase basal calcium level in nonadherent (**E**, control *n* = 14, sAβ *n* = 17) or adherent microglia (**F**, control *n* = 16, sAβ *n* = 14). ***p < 0.001.

Distinct from LPS-induced calcium response, freshly solubilized Aβ (termed as sAβ) induced a pronounced transient calcium response or, interestingly, an oscillatory calcium response (Figure 2A). Calcium response to freshly solubilized Aβ was detected in majority of cells while 90% of these responses were oscillatory by nature, and the amplitude was far greater than induced by LPS (Figures 2B,C). Surprisingly, the amplitude of calcium response induced by Aβ decayed after the solubilization event time-dependently (Figure 2B). Also, the occurrence of calcium response decreased after the solubilization event (Figure 2C). As a comparison, the occurrence of calcium responses to ATP in the same cells remained constant and did not decrease time-dependently (Figure 2C). Prefibrillized Aβ (termed as fibrillar Aβ, fAβ) induced a small calcium response (in 50% of the stimulated cells, with Δ 340/380 ratio value of 190) resembling the time-dependent decayed calcium response of sAβ (Figure 2D). As seen in Figure 2A, calcium oscillations induced by freshly solubilized Aβ persisted even after removal of Aβ stimulus. Therefore, calcium responses induced by MCSF could not be reliably determined after Aβ stimulus since they were mainly covered by oscillatory calcium responses induced by sAβ. Also, the magnitudes of ATP-induced calcium responses could not be reliable determined since they were partly masked by persistent oscillatory response induced by sAβ. Aβ may utilize same calcium signaling pathways thus underestimating the magnitude of ATP response. To be noted, ATP did not induce oscillatory calcium signaling but calcium transients.

To examine whether sAβ-induced calcium oscillations occur in other mononuclear phagocytes, we performed calcium imaging for primary microglia, collected from mixed astrocyte/microglia culture as nonadherent microglia cells above the astrocyte layer and as adherent microglia cells growing below astrocyte layer. Microglia had less stable basal intracellular calcium level (Figures 2E,F) compared to monocytic cells (Figures 1C, 2A,D). Microglia produced spontaneous calcium transients without stimulus (Figures 2E,F), which interfered with further detection of possible sAβ-induced responses. However, we did not detect a clear oscillatory pattern in microglia (Figures 2E,F) similar to that observed in monocytic cells. In addition, calcium levels were not further increased after sAβ stimulus, compared to basal levels without stimulus (Figures 2E,F). Both microglia types produced prominent calcium response to ATP (Figures 2E,F), served as a control stimulus. In general, it is very difficult to maintain microglia in resting state *in vitro*; any change in their culture environment tends to transform them into activated state. This may cause difficulties in sensitive measurements as calcium signaling.

To test whether sAβ-induced calcium oscillations could be reduced with anti-inflammatory treatment, monocytic cells were preincubated with neuroprotective and neuroinflammatory drug minocycline (Tikka et al., 2001; Malm et al., 2008). One day or immediate preincubation with minocycline (5 μM), however, had no effect on sAβ-induced calcium oscillations (data not shown). Taken together, the data suggest that sAβ induces oscillatory, persistent calcium signaling in monocytic cells. We next aimed to decipher how calcium responses induced by Aβ affected monocytic cell activation and viability.

### Aβ Species Induce Anti-Inflammatory but No Proinflammatory Activation of Monocytic Cells, Contrary To Primary Microglia

**Figure 3:**
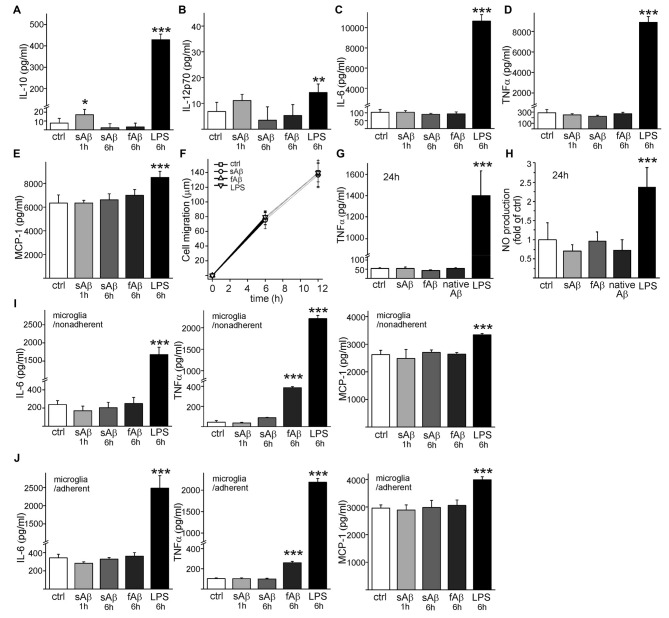
**sAβ induces interleukin-10 (IL-10) production but no proinflammatory cytokine or nitric oxide (NO) production in monocytic cells.** Monocytic cells were stimulated with synthetic 10 μM sAβ or fAβ or 100 ng/ml LPS upto 6 h. Cytokine production was analyzed from cell culture medium with cytometric bead array utilizing flow cytometry. SAβ induced a transient production of IL-10 (**A**, *n* = 4–8) but no significant production of IL-12 was detected (**B**, *n* = 4–8). Aβ did not cause any production of pro-inflammatory cytokine IL-6 of Tumor necrosis factor α (TNFα) (**C,D**, *n* = 4–8). As an annotation, interferon-γ was under detectable level in monocytic cells. While monocyte chemoattractant protein-1 (MCP-1) production in monocytic cells was high (**E**, *n* = 4–8), Aβ or LPS did not affect cell migration as determined for 12 h (**F**, *n* = 8) when monitoring the cell trajectory length in automated cell culture and analysis system (Cell IQ). When confirming proinflammatory cytokine production with ELISA, TNFα was neither produced if monocytic cells were incubated on top of brain sections from aged APdE9 mice containing native Aβ deposits nor in the presence of synthetic Aβ (**G**, *n* = 4). Again, LPS (50 ng/ml) induced high production of TNFα (**G**, *p* < 0.001, *n* = 6). Similarly, synthetic Aβ (*n* = 8) or native Aβ deposits (*n* = 4) did not induce NO production like LPS (**H**, *p* < 0.001, *n* = 8). Analyzed with cytometric bead array, 6 h incubation with fAβ induced production of TNFα in primary microglia (**I,J**, *p* < 0.001, *n* = 4). Interferon-γ, as well as IL-10 and IL-12 were under detectable levels in microglia. LPS served as a positive control for cytokine production. *p < 0.05, **p < 0.01, ***p < 0.001.

Interestingly, sAβ induced a small, significant production of anti-inflammatory cytokine IL-10 (*p* < 0.05, Figure 3A) which was transient and decayed until 6 h incubation. There was no significant production of IL-12 (active heterodimer IL-12p70) in response to sAβ (*p* = 0.06, Figure 3B). Neither synthetic sAβ nor fAβ caused any production of pro-inflammatory cytokines IL-6 (Figure 3C) or TNFα (Figure 3D). Monocytic cells had high production of monocyte chemoattractant protein-1 (MCP-1), but it was not affected by sAβ (Figure 3E). LPS, on the other hand, increased production of all aforementioned cytokines (Figures 3A–E). Cytokine production did not, however, affect cell migration (Figure 3F). Furthermore, when monocytic cells were incubated on top of brain sections from APdE9 mice containing substantial amount of native Aβ deposits as well as inflammatory environment present in the AD mouse brain, no TNFα production was observed (Figure 3G). Synthetic Aβ or native Aβ deposits induced no NO production while LPS induced a notable production of NO (Figure 3H). This is in line with our previous results where we have shown these HSC-derived monocytic cells to produce inflammatory cytokine TNFα and release NO after inflammatory stimulation with LPS (Magga et al., 2012).

In comparison, we also studied how Aβ species activate primary microglia. Unlike monocytic cells, the production of IL-10 and IL-12p70 cytokines in microglia remained under detectable level. Aβ species did not induce any anti-inflammatory cytokine production in microglia. LPS induced production of IL-10 (42 ± 6 pg/ml in nonadherent, 33 ± 2 pg/ml in adherent microglia, respectively) but no IL-12p70. sAβ did not produce any inflammatory activation in microglia, but fAβ induced production of proinflammatory cytokine TNFα in microglia (Figures 3I,J). LPS increased production of IL-6, TNFα and MCP-1 in microglia as expected (Figures 3I,J).

### Aβ Does Not Induce ROS Production But Affects Monocytic Cell Viability Species-Dependently

**Figure 4:**
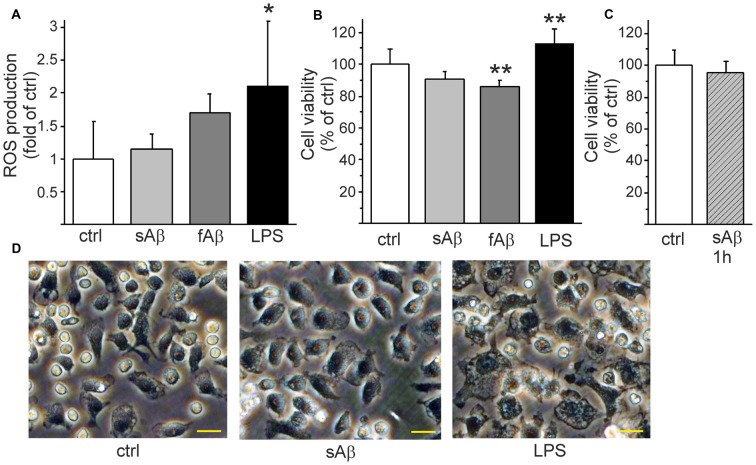
**Aβ affects monocytic cell viability in aggregation stage-dependent manner.** LPS induced a pronounced production of reactive oxygen species (ROS) (**A**, *p* < 0.05, *n* = 7). LPS increased cell proliferation/survival (**B**, *p* < 0.01, *n* = 8) while synthetic fAβ slightly reduced cell viability (**B**, *p* < 0.01, *n* = 8). When monocytic cells were stimulated with freshly solubilized sAβ for 1 h, then washed and incubated another 23 h in the absence of Aβ to mimic the short stimulation which induced calcium oscillations there was no reduction of cell viability (**C**, *n* = 8). sAβ did not change monocytic cell morphology as observed after LPS stimulation **(D)** as pictured here 24 h after stimulation. Scale bar 20 μm. *p < 0.05, **p < 0.01.

Since sAβ induced calcium oscillations and anti-inflammatory cytokine production but did not induce pro-inflammatory cytokine or NO production, we further investigated whether Aβ induces ROS production or affects monocytic cell viability. Aβ did not induce ROS production while LPS induced a pronounced production of ROS (Figure 4A). Nevertheless, fAβ treatment triggered a slightly reduced cell viability (Figure 4B). Although LPS induced pro-inflammatory activation and clear ROS production, the cell viability was rather increased (Figure 4B). We have previously demonstrated that HSC-derived monocytic cell growth is greatly dependent on MCSF as a growth factor, or on the inflammatory stimulus LPS (Magga et al., 2012).

Finally, we investigated how the persistent oscillatory response of freshly solubilized sAβ (Figure 2A) affects monocytic cell function. We stimulated the cells with freshly solubilized sAβ for 1 h, then washed the cells and changed the medium to exclude further stimulation with aggregating Aβ. When analyzed 24 h later, short stimulation with freshly solubilized sAβ, capable of inducing persistent calcium oscillations (Figure 2A) was not found to reduce cell viability (Figure 4C). While LPS induced the classical ameboid phenotype, sAβ did not change cell morphology into ameboid inflammatory phenotype (Figure 4D). Taken together, sAβ did not induce pro-inflammatory cytokine, NO or ROS production and did not affect cell viability but induced a production of anti-inflammatory cytokine. This finding suggests that the oscillatory calcium response induced by freshly solubilized sAβ is not a detrimental stimulus to monocytic cells.

### Monocytic Cells Retain Their Characteristics as Phagocytic Cells Towards Native Aβ Deposits *Ex Vivo*

**Figure 5:**
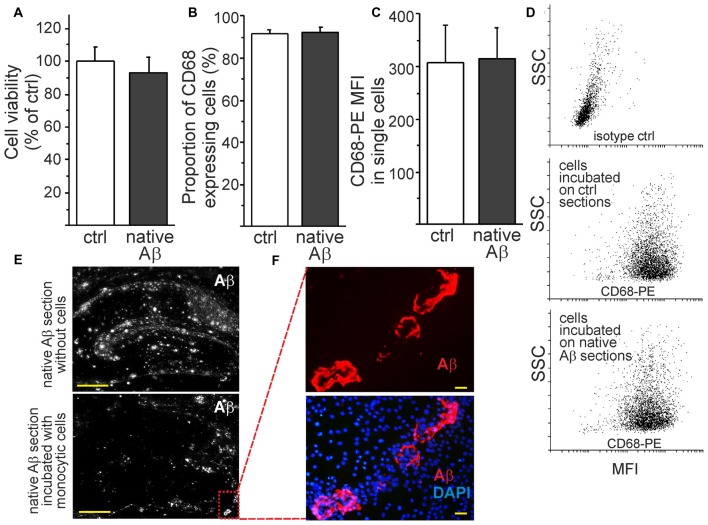
**Monocytic cells retain their viability and phagocytic activity within native Aβ deposits *ex vivo*.** The incubation of monocytic cells with native Aβ deposits in hippocampal sections obtained from aged APdE9 mice did not affect cell viability (**A**, *n* = 8). Monocytic cells also retained their characteristics as phagocytes since no difference in the percentage of CD68^+^ expressing cells (**B**, *n* = 6) or the expression level of CD68 (mean fluorescent intensity (MFI)) in single cells were observed (**C**, *n* = 6). This was analyzed by flow cytometry after collecting the cells incubated on top of control (wt) and native Aβ (APdE9) mouse hippocampal sections **(D)**. Monocytic cells phagocytosed both diffuse and hard Aβ plaques **(E)**. Monocytic cells gathered around vascular Aβ plaques but were not observed to phagocytose them despite of high cell concentration **(F)**. Scale bar 20 μm **(F)**, 500 μm **(E)**.

To investigate the monocytic function in the presence of native Aβ deposits, we cultivated the cells on top of brain sections obtained from aged APdE9 and wild type mice. The environment rich in native Aβ deposits did not affect cell viability (Figure 5A). Monocytic cells retained also their characteristics as phagocytes. This was evidenced by flow cytometry analysis that showed no difference in the expression of lysosomal and phagocytosis marker CD68 on cells incubated on top of brain sections prepared from aged wild type or APdE9 mice (Figures 5B–D). These findings are-in line with our previous report on the potential of monocytic cells to internalize or clear Aβ *in vitro*, *ex vivo* and *in vivo* (Magga et al., 2012). Furthermore, in the areas of high rate of phagocytosis, monocytic cells tended to eventually phagocytose both diffuse and hard Aβ plaques, leaving vascular Aβ plaques intact (Figures 5E,F). In *ex vivo* assay, monocytic cells are exposed to both insoluble and sAβ during cell attachment and phagocytosis, the level of sAβ42 (19.1 ± 12.1 pg/ml) roughly corresponding to concentrations of Aβ42 in human plasma.

### Monocytic Cells Reduce Congophilic Aβ Plaques *In Vivo*

**Figure 6:**
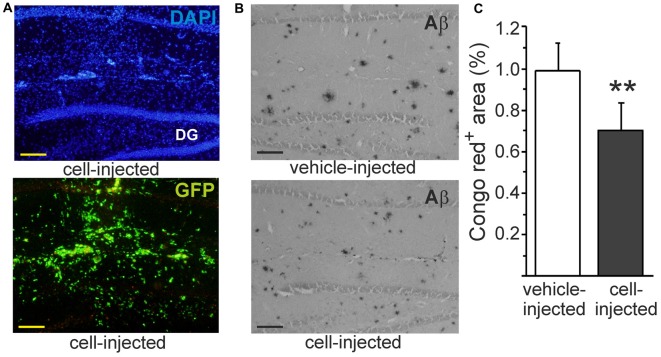
**Intrahippocampally transplanted monocytic cells reduce congophilic Aβ plaques in Alzheimer’s disease (AD) mouse model *in vivo*.** Monocytic cells obtained from green fluorescent protein (GFP) positive transgenic mouse were transplanted unilaterally into the hippocampus **(A)** using contralateral vehicle-injection as a control. Hard Aβ plaques were identified with Congo red stain **(B)**. Monocytic cells reduced congophilic Aβ plaques at the site of cell injection (**C**, *p* < 0.01, *n* = 5). Scale bar 100 μm. DG, dentate gyrus. GFP and Congo red were determined from consequtive sections and pictured from the same site of the hippocampus. **p < 0.01.

Our previous studies have shown that intrahippocampally transplanted monocytic cells survive well in APdE9 mouse brain and reduce Aβ deposits as detected within pan-Aβ immunoreactive area (Magga et al., 2012). In line with the earlier study, transplanted monocytic cells detected by their green fluorescent protein (GFP) fluorescence (Figure 6A) were observed in the hippocampus 4 days after transplantation. In addition to reduction of diffuse Aβ plaques in the hippocampus (Magga et al., 2012), transplanted monocytic cells also reduced Congo red positive Aβ plaques in the areas occupied by the cells (Figures 6B,C). When Aβ was quantified from the areas devoid of transplanted cells, there was no reduction of Congo red positive Aβ plaques. This finding further suggests that monocytic cells reduce Aβ plaques by phagocytosis and emphasizes the fact that monocytic cells should be drawn into the lesion site at sufficient quantity.

## Discussion

First, the role of monocytic cells in AD pathology remains unresolved although recent studies have provided insight into this issue. CCR2, a chemokine receptor, was shown to be a prerequisite for microglia accumulation in brain, to restrict AD pathology (El Khoury et al., 2007). More specifically, CCR2 deficiency in bone marrow cells exacerbated AD pathology including mnesic deficits (Naert and Rivest, 2012). This suggests CCR2 to play an important role for proper monocyte/microglia function in AD. Mouse monocytes exist as two main subsets: inflammatory CCR2^+^CX3CR1^low^Ly6C^+/high^ and resident CCR2^-^CX3CR1^high^Ly6C^-/low^ monocytes. These monocyte subsets display different migration properties and function in inflammation and steady state conditions (Auffray et al., 2009). Although inflammatory monocytes are thought to rapidly infiltrate into inflamed brain and participate in functions such as inflammatory cytokine secretion and phagocytosis (reviewed in Malm et al., 2010), there is recent evidence that Ly6C^low^ patrolling monocytes also play a role in restricting Aβ (Michaud et al., 2013). To be able to understand how monocytic cells become activated and react in AD brain, it is important to clarify their responses to specific stimuli. We aimed to challenge the idea of Aβ acting as a classical pro-inflammatory stimulus and investigated the effects of different forms of Aβ on HSC-derived monocytic cells. As we show here, different forms of Aβ contribute to separate cellular responses thereby possibly regulating subcellular events and ultimately, cell functions including phagocytosis.

We first aimed to decipher cellular responses of monocytic cells to a known inflammatory stimulus. The most frequently used model agent to study inflammatory cell activation is the Gram-negative bacterial endotoxin LPS. It activates macrophages and microglia through stimulating Toll-like receptor 4, although recent studies suggest that alternative or even Toll-like receptor 4 independent signaling pathways are triggered when cells are exposed to LPS (Kayagaki et al., 2013; Qu et al., 2015). We detected a classical inflammatory cytokine and NO production, well-known responses to LPS stimulation in cells of the immune system. LPS has been shown to increase intracellular calcium level in primary microglia cells, which is associated with LPS-induced inflammatory activation such as secretion of cytokines and NO. Moreover, increased intracellular calcium level has been reported to be accompanied with decreased responsiveness to external purinergic stimulation (Hoffmann et al., 2003). In line with these previous observations, we detected a minor increase in intracellular calcium levels in response to LPS, which was accompanied with an immediate increase in cell protrusion motility. On the contrary, LPS-stimulation did not reduce cellular response to purinergic stimulus in our monocytic cell model. Instead, LPS reduced growth factor—induced oscillatory calcium response. This indicates that although the calcium signaling patterns induced by LPS are somewhat similar in general, subtle cellular responses to LPS may vary between different myeloid subtypes. This is probably dependent on the cellular signaling profile of myeloid cell subsets.

MCSF has been shown to induce calcium oscillations in a microglia cell line. It was suggested further that MCSF induces calcium-binding protein Iba-1 to play a role in membrane ruffling and phagocytosis (Ohsawa et al., 2000). In line with this, we detected MCSF to induce calcium oscillations. HSC-derived monocytic cells also express Iba-1 as we have shown earlier (Magga et al., 2012). In general, MCSF signaling is a critical regulator of mononuclear phagocytic cell survival, differentiation, development and chemotaxis (Nayak et al., 2014).

Remarkably, our monocytic cells responded to freshly solubilized synthetic Aβ by inducing persistent calcium oscillations. Interestingly, oscillatory responses to Aβ diminished and eventually vanished after Aβ solubilization time-dependently, within 60–90 min after solubilization. Since calcium oscillations decayed time-dependently in the course of Aβ aggregation, we concluded that oscillatory calcium responses are dependent on specific Aβ aggregation stage, i.e., monomeric and/or small oligomeric Aβ. Aβ stimulus that induced persistent calcium oscillations, induced a transient production of anti-inflammatory cytokine but did not induce any pro-inflammatory response in terms of ROS, NO or cytokine production, nor reduced monocytic cell viability. Instead, monocytic cells retained their phagocytic phenotype in native Aβ-laden environment when all forms of Aβ were present. Importantly, intrahippocampally transplanted monocytic cells reduced Congo red positive fibrillar Aβ plaques from AD mouse brain *in vivo*. On the contrary, microglial cells obtained from aged AD mice have been shown to have defective Aβ clearance pathways (Hickman et al., 2008; von Bernhardi et al., 2015). Moreover, an inverse correlation between pro-inflammatory cytokine production and Aβ clearance was demonstrated to microglia (Hickman et al., 2008).

In non-excitable cells, the main way to evoke oscillations is through receptor-mediated activation of phospholipase C, creation of diacylglycerol, and subsequent formation of second messenger inositol 1,4,5-trisphosphate and periodical release of calcium from endoplasmic reticulum calcium store. Oscillations are usually accompanied with activation of store-operated calcium channels and extracellular calcium influx to maintain proper levels for calcium cycle. Oscillatory calcium signaling has been associated with several cellular events including secretion, metabolism, cell maturation and differentiation, chemotaxis and induction of gene expression. Thus, oscillation-induced cellular events range from seconds over hours to days. By oscillations, a cell avoids toxic effects that arise from a constant calcium elevation. As another benefit, oscillatory signals support long-lasting cellular responses because of lower desensitization of calcium-dependent responses that can occur during a prolonged constant calcium signal, as reviewed in Parekh (2011). Interestingly, in macrophages, oxidized LDL has been reported to induce calcium oscillations which were suggested to promote antiapoptotic pathways (Chen et al., 2009). In addition, calcium oscillations are known to serve as cell survival signals in T-cells (Harr and Distelhorst, 2010).

Disturbances in neuronal calcium signaling may play a crucial role in the development of AD (Berridge, 2010), mostly in terms of amyloid metabolism and action of presenilins. The role of calcium signaling in glial cells or mononuclear phagocytes is inadequately understood. In the adult brain, blood-derived monocytes have the potential to repopulate myeloid niche and calcium transients observed in engrafted monocytes are very similar to that of surveillant resident microglia (Varvel et al., 2012). Most of the studies concerning the actions of mononuclear phagocytes however, are conducted with microglia. There are many ligands which could induce calcium signaling in microglia. These include chemokines, vasoactive peptides, thrombin, growth factors and transmitters such as acetylcholine, glutamate and noradrenaline (Möller, 2002; Kettenmann et al., 2011). Several studies have shown elevation of intracellular calcium in microglia/monocyte cells in response to Aβ (Korotzer et al., 1995; Combs et al., 1999; Cui et al., 2002; Moon et al., 2008; Sanz et al., 2009). While none of these studies reported oscillatory calcium signaling, these studies suggested that Aβ-induced calcium signaling promotes pro-inflammatory cellular response which we did not observe after stimulation with any type of synthetic or native Aβ deposits in our HSC-derived monocytic cells. Besides cell type difference, this discrepancy might also be explained by the fact that LPS is a frequent and functionally significant contaminant in many commercial-grade preparations of peptides or other substances commonly used in research on microglial activation (Weinstein et al., 2008). In addition, the aggregation and thus the effects of Aβ may be different if Aβ is dissolved into organic solvent or aqueous buffer.

Microglia may produce also ROS, reactive nitrogen species and secreted cytokines in response to Aβ (Moon et al., 2008; Querfurth and LaFerla, 2010). On the other hand, calcium signaling pathways may interact with other cellular signaling systems such as ROS (Görlach et al., 2015), indicating that disturbed calcium signaling may result in altered ROS production and thus harmful activation of cells. Importantly, perturbations in calcium signaling have been reported in microglia obtained from AD patients. Receptor for advanced glycation end products, a transmembrane receptor of immunoglobulin family, and G-protein coupled formyl peptide receptor found on microglia can both bind Aβ, resulting in enhanced neuroinflammation and acceleration of AD pathology via altered calcium signaling (Nayak et al., 2014). Similar perturbations in cellular calcium were also obtained when treating primary microglia with fibrillar Aβ: elevations of basal calcium level accompanied with reduced amplitude to ligands such as ATP indicating decreased activity of store-operated calcium channels (McLarnon et al., 2005). In two-photon imaging *in vivo*, cortical microglia were rather silent in terms of their calcium signaling but they responded with large calcium transients to focal damage of an individual neuron in their vicinity (Eichhoff et al., 2011). Interestingly, calcium transients evoked by minor cell or tissue damages in the vicinity of microglial cells may be especially frequent near plaques, as reflected by profound hyperactivity of plaque-associated microglia (Brawek et al., 2014). Similar persistent calcium oscillations in response to Aβ what we observed have been previously reported in astrocytes where they were accompanied with neurotoxic effects in hippocampal coculture (Abramov et al., 2003). However, neurotoxicity was studied within 24 h of Aβ incubation, while immediate cellular effects other than calcium signaling were not assessed. In summary, Aβ-induced calcium signals are thought to be associated with harmful activation of cells by evoking pro-inflammatory pathways, which are generally thought to be associated with neurotoxicity. In the present study, we did not observe sAβ-evoked calcium oscillations to induce activation of pro-inflammatory pathways in monocytic cells.

Activation of metabotropic glutamate receptor mGlu5 is known to induce calcium oscillations (Nash et al., 2001). mGlu5 was found to negatively regulate the release of microglia-associated inflammatory factors and related neurotoxicity (Byrnes et al., 2009; Farso et al., 2009). There is some evidence that mGlu5 could function as a receptor or co-receptor for Aβ (Kumar et al., 2015), although Aβ-mGlu5 interaction specifically in microglia is not known. It remains to be clarified in future experiments whether sAβ-induced calcium oscillations we observed in monocytic cells: (i) enquire mGlu5 receptors; (ii) serve as a signal to induce several anti-inflammatory pathways; and (iii) prime monocytic cells for phagocytosis.

It has been reported on astrocytes that effects of Aβ and pro-inflammatory factors on the cytosolic calcium dynamics are different. mGluR-mediated calcium signaling as well as store-operated calcium entry were augmented in response to Aβ while opposite changes were observed when astrocytes were treated with pro-inflammatory agents TNFα, IL1β and LPS (Ronco et al., 2014). In line with our present study, it could be concluded that Aβ cannot be regarded as a traditional pro-inflammatory agent in monocytic cells; to the contrary, Aβ employs alternative pathways to affect the state and function of these cells.

## Conclusion

In this study, we show that Aβ induces specific calcium signaling in HSC-derived monocytic cells which does not lead to harmful inflammatory activation nor reduces viability of these cells. To our knowledge, this is the first study to show an oscillatory signal in response to sAβ in mononuclear phagocytes. When further characterized, this signaling pathway may introduce a novel therapeutic target in mononuclear phagocytic cells. Although its purpose is currently unknown, this signal may promote phagocytosis or prime the cells to encounter the possible threat of coming excessive Aβ load.

Our study also suggests that HSC-derived monocytic cells retain their viability and function in the presence of native Aβ deposits while reducing Aβ burden even by removal of hard Aβ plaques. This further affirms that HSC-derived monocytic cells could possibly be used as cell-based therapeutics in the treatment of AD. Since these cells could be genetically modified before their differentiation into monocytic cells, the further challenge is to program the cells to enhance their proper infiltration into the brain parenchyma.

## Author Contributions

ES conducted *in vivo* and *in vitro* studies with monocytic cells and analyzed *in vivo* results. TM participated in *in vivo* studies. HK, RHH, CG-T, SW and RG participated in *in vitro* studies with monocytic cells and microglia, and analysis and interpretation of data. JM performed *in vitro* studies and drafted the manuscript. JK and JM designed the study. TM and JK revised the manuscript. All authors have read and approved the final manuscript.

## Funding

This study was supported by Academy of Finland, The Finnish Funding Agency for Innovation TEKES and Sigrid Juselius Foundation.

## Conflict of Interest Statement

The authors declare that the research was conducted in the absence of any commercial or financial relationships that could be construed as a potential conflict of interest.

## Abbreviations

AD: Alzheimer’s disease
Aβ: Amyloid β
APdE9: human amyloid precursor protein APP695 Swedish mutation and human presenilin-1 deletion in exon 9 mouse line
ATP: adenosine triphosphate
CNS: central nervous system
fAβ: fibrillar Amyloid β
GFP: green fluorescent protein
ELISA: enzyme-linked immunosorbent assay
HSC: hematopoietic stem cell
IL: interleukin
LPS: lipopolysaccharide
MCP-1: monocyte chemoattractant protein-1
MCSF: macrophage colony stimulating factor
NO: nitric oxide
ROS: reactive oxygen species
sAβ: soluble Amyloid β
TNFα: tumor necrosis factor α.
